# Genetic Variants of Diabetes Risk and Incident Cardiovascular Events in Chronic Coronary Artery Disease

**DOI:** 10.1371/journal.pone.0016341

**Published:** 2011-01-20

**Authors:** André Gustavo P. Sousa, Neuza H. Lopes, Whady A. Hueb, José Eduardo Krieger, Alexandre C. Pereira

**Affiliations:** 1 Laboratory of Genetics and Molecular Cardiology, Medical School, Heart Institute, University of São Paulo, São Paulo, Brazil; 2 Clinical Medicine Department, Federal University of Rio Grande do Norte, Natal, Brazil; 3 Coronary Artery Disease Service, Medical School, Heart Institute, University of São Paulo, São Paulo, Brazil; University of Tor Vergata, Italy

## Abstract

**Objective:**

To determine whether information from genetic risk variants for diabetes is associated with cardiovascular events incidence.

**Methods:**

From the about 30 known genes associated with diabetes, we genotyped single-nucleotide polymorphisms at the 10 loci most associated with type-2 diabetes in 425 subjects from the MASS-II Study, a randomized study in patients with multi-vessel coronary artery disease. The combined genetic information was evaluated by number of risk alleles for diabetes. Performance of genetic models relative to major cardiovascular events incidence was analyzed through Kaplan-Meier curve comparison and Cox Hazard Models and the discriminatory ability of models was assessed for cardiovascular events by calculating the area under the ROC curve.

**Results:**

Genetic information was able to predict 5-year incidence of major cardiovascular events and overall-mortality in non-diabetic individuals, even after adjustment for potential confounders including fasting glycemia. Non-diabetic individuals with high genetic risk had a similar incidence of events then diabetic individuals (cumulative hazard of 33.0 *versus* 35.1% of diabetic subjects). The addition of combined genetic information to clinical predictors significantly improved the AUC for cardiovascular events incidence (AUC = 0.641 versus 0.610).

**Conclusions:**

Combined information of genetic variants for diabetes risk is associated to major cardiovascular events incidence, including overall mortality, in non-diabetic individuals with coronary artery disease.

**Clinical Trial Registration Information:**

Medicine, Angioplasty, or Surgery Study (MASS II). Unique identifier: ISRCTN66068876 URL.

## Introduction

Type-2 diabetes mellitus (T2DM) is a common disease present in about 200 million individuals around the world and its prevalence has been increasing [1]. Genome-wide association studies (GWASs) have identified about 30 susceptibility genes for T2DM in different populations [2], [3], [4], [5], [6], [7], [8], of which the most important (based on estimated size effect) are *PPARG*, *SLC30A8*, *HHEX*, *TCF7L2*, *KCNJ11*, *IGFBP-2*, *CDKAL1*, *CDKN2A/B* and *FTO*. Nevertheless, these polymorphisms have, individually, only small effects on T2DM risk, with odds ratios around 1.3. Considering that genetic susceptibility for complex diseases such as T2DM is caused by multiple genetic variants, predictive tools based on information from a single genetic polymorphism will be of limited value [9]. Accordingly, studies have found that the predictive value for T2DM diagnosis can be improved by combining multiple common low-risk variants [9], [10], [11], [12], [13], [14], [15]. The importance of predicting the risk of an individual to present diabetes is related to the possibility of early diagnosis and the potential avoidance of T2DM-related complications, particularly cardiovascular disease. Despite this fact, fewer studies have concentrated efforts in determining whether knowledge of T2DM-associated SNPs in individuals with established cardiovascular disease can be of any good. Recently, a study have shown the association of polymorphism rs7903146 of *TCF7L2* gene and cardiovascular events incidence [16], but it did not assess whether the combination of multiple risk variants beyond *TCF7L2* is useful in predicting cardiovascular events.

In this study, we tested whether the combination of multiple genetic risk variants known to predict T2DM can be used to predict cardiovascular events in individuals with chronic coronary artery disease.

## Methods

### Study population

The MASS II trial was a prospective, randomized and controlled clinical trial that was designed to compare medical treatment, percutaneous coronary intervention (PCI), and coronary artery bypass graft (CABG) surgery in patients with stable multi-vessel CAD and preserved left ventricular function. Briefly, individuals who were indicated for myocardial revascularization were evaluated from May, 1995 to May, 2000 and were randomly assigned to one of the three therapeutic groups. The inclusion criteria were symptomatic multi-vessel coronary disease, preserved left ventricular function, and the presence of coronary lesions (70% of stenosis) amenable both to angioplasty and surgery. Enrolled patients were followed up for five years and were assessed for cardiovascular events (overall-mortality, myocardial infarction, PCI, CABG) or the combination of them. The diagnosis of diabetes mellitus was based on previous treatment for diabetes or ADA criteria [17]. Individuals that did not meet criteria for diabetes according ADA were included in the non-diabetic group at baseline. Other studies that used this same population for similar analysis have been previously published [18], [19], [20], [21].

We genotyped single-nucleotide polymorphisms (SNP) at 10 loci associated with type-2 diabetes in 425 subjects from the MASS-II Study and a 5-years follow-up was performed. Other 186 individuals who were initially randomized were excluded because incomplete genotypic information from all studied genetic markers was available. A comparison of baseline characteristics between included and excluded individuals was performed and there were no significant differences between them (See Table S1 in Supplementary Material). All subjects gave written informed consent, and the Ethics Committee of the Heart Institute of the University of São Paulo-Brazil approved the study. All procedures were performed in accordance with the Helsinki Declaration of 1975 (revised 1983).

### Selection of SNPs, risk alleles, and genotyping

A large-scale meta-analysis performed for T2DM in populations of European ancestry [4] reported consistent associations between T2DM and ten SNPs: *KCNJ11* rs5219, *PPARG* rs1801282, *TCF7L2* rs7903146, *SLC30A8* rs13266634, *HHEX* rs1111875, *CDKAL1* rs7754840, *IGFBP2* rs4402960, *FTO* rs8050136, *CDKN2A/B* rs10811661 and rs9300039. We selected these genetic markers for the present study.

Genomic DNA was extracted from peripheral blood leukocytes by means of a standard salting-out procedure. Genotyping assays were run as submicroliter PCR-based assays on array tape, which is a continuous plastic tape that is used in conjunction with a flexible configuration of dispensing, pipetting, sealing and detection modules manufactured by Douglas Global Array. Thermocycling was performed in sealed master tape in aqueous immersion in the thermocycler. A modified allele-specific PCR assay as described by Myakishev [22] was used. We obtained an average genotyping success rate of more than 95% and an average genotyping accuracy of 100% by regenotyping 32 samples. All SNPs were in Hardy–Weinberg equilibrium (P>0.001).

For the analysis of the effects of the combined genetic variants, we used an additive genetic model, i.e., the total number of risk alleles (each individual could have 0,1, or 2 alleles, for a total of 20, in 10 genes), which were significantly associated with diabetes. A clinical model that contained only clinical variables associated with mortality and re-infartion in MASS-II study (age, arterial hypertension and previous myocardial infarction) was built by the sum of Cox regression coefficient values of each variable multiplied by 10, and rounded to the nearest integer (See Table S2 with these values in Supplemental Material). The incremental value of combining genetic information to the clinical risk factors model was also evaluated.

### Statistical Analysis

Chi-square tests, t-tests, and analysis of variance were used for baseline comparisons. For multiple comparisons, a more restrictive significance threshold for p value was used by a Bonferroni's correction for multiple comparisons. Long-term survival comparisons were conducted for the entire study group and for the subgroups of diabetic and non-diabetic patients at baseline. Cox regression was used to estimate the prospective association of these variables with each of the endpoints (death, myocardial infarction, and recurrent ischemia requiring revascularization), as well as the combined endpoint after 5-year follow-up. Survival curves were calculated with the Kaplan-Meier method, and differences between the curves were evaluated with the log-rank statistic. In order to evaluate the models performance, a receiver operating characteristic (ROC) curve was built and the area under ROC curve (AUC or C statistics) was used to measure the discriminative power. C statistic for the clinical model and the genetic information added to the clinical model were compared by using the ROCKIT software (http://www-radiology.uchicago.edu/krl/KRL_ROC/software_index6.htm). Statistical analyses were performed with the use of SPSS software, version 13.0.

## Results

In the MASS-II population, a total of 425 individuals were genotyped for all of the ten studied SNPs, from whom 134 had diabetes (31.5%). Clinical characteristics of participants at baseline are provided in Table 1 and characteristics of the 10 risk loci are shown in Supplemental Material (Table S3). Diabetic and non-diabetic subjects differed in relation to hypertension prevalence. Therefore, all further tests were corrected for this variable as a possible confounder. Only *TCF7L2* gene was singly associated with T2DM in preliminary analysis, however, after correction for multiple comparisons and considering a p value more restrictive (p<0.005), *TCF7L2* polymorphism did not reach statistical significance. Initially, we evaluated the association between the combination of risk alleles and the diagnosis of diabetes in this population and this association was significant (See Supplementay Material, Tables S4 and S5).

**Table 1:** Clinical characteristics of diabetic individuals and non-diabetics from MASS-II Study.

	Diabetes (n = 134)	Non-diabetic (n = 291)	P value
Age (years)	60.40±8.82	59.31±9.36	0.256
Gender (male %)	63.4%	69.1%	0.250
BMI (kg/m^2^)	27.68±4.44	26.98±4.00	0.109
Total cholesterol (mg/dl)	219.23±50.56	226.31±50.27	0.181
HDL-cholesterol (mg/dl)	38.19±11.27	36.76±9.86	0.200
LDL-cholesterol	143.78±45.67	150.93±44.34	0.141
Triglycerides (mg/dl)	204.78±145.07	190.26±105.24	0.247
Hypertension (%)	67.9%	54.3%	0.008
Smoking (%)	28.4%	35.1%	0.173
Previous MI (%)	42.5%	46.7%	0.419
Three-vessel disease (%)	56.7%	54.6%	0.689

Data are shown in mean ± SD or percentage.

**Figure 1:**
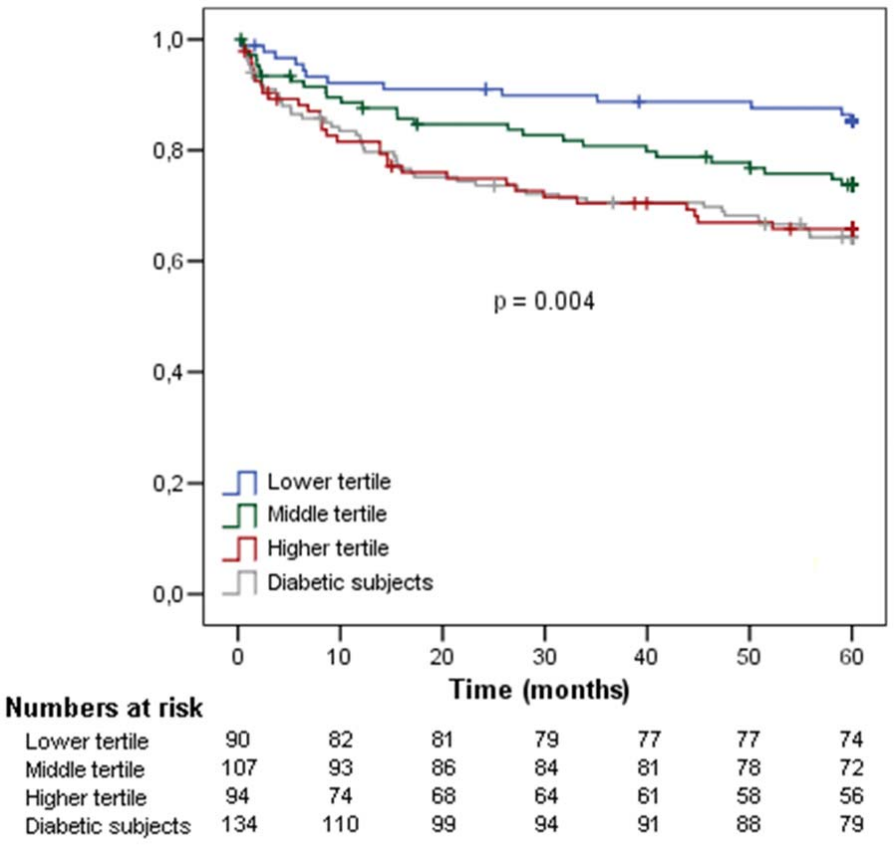
Kaplan-Meier Curves of combined T2DM risk alleles and composite cardiovascular end-point in non-diabetic individuals separated by groups according to number of risk alleles tertiles and diabetic subjects after 5 years of follow-up. Note diabetic individuals' curves are similar to higher tertiles' curves. P values to pairwise comparisons: Lower *vs* middle: p = 0.052; lower *vs* higher: p = 0.002; lower *vs* diabetics: p = 0.001; middle *vs* higher: p = 0.178; middle *vs* diabetics: p = 0.101; higher *vs* diabetics: p = 0.870.

In order to evaluate if T2DM risk alleles were associated with cardiovascular events incidence in the MASS-II population, we performed a division of individuals in three groups based on the number of risk alleles tertiles: lower risk (0 to 11 risk alleles), middle (12 or 13 alleles) and higher (14 or more risk alleles). Although diabetic individuals had a higher incidence of cardiovascular events than non-diabetics (35.1% *vs* 24.4, p = 0.022), it was observed that genetic tertiles were not associated with the composite end point of cardiovascular events (cardiac death, myocardial infarction, and refractory angina requiring revascularization or new percutaneous coronary intervention – PCI) in individuals with already established diabetes. Interestingly, however, individuals with higher number of alleles were associated with a significantly higher incidence of cardiovascular events in non-diabetic individuals (Kaplan-Meier curve analysis shown in Figure 1). In addition, there were no significant differences between the estimates of the probability of survival from diabetic individuals and non-diabetic individuals in the higher genetic risk group (p value>0.4). After 5-years of follow-up, diabetic individuals presented a cumulative incidence of the combined end-point of 35.1% against non-diabetic individuals in the third tertile risk group that presented 33.0% (Table 2). To corroborate these findings, there was significant interaction between diabetes and risk alleles for combined end-points (p value = 0.029). These data suggest that non-diabetic individuals whom have high number of risk alleles have similar outcomes as individuals with already established diabetes.

**Table 2:** Number of composite cardiovascular events and cumulative hazard in diabetic subjects and non-diabetic subjects according to number of risk alleles tertiles.

	Number of events	Cumulative Hazard (%)	P value	P value for interaction
Non-diabetic individuals				
Lower tertileMiddle tertileHigher tertile	132731	14.425.233.0	0.004*	0.029§
Diabetic individuals	47	35.1	0.022‡	

(*) P value for comparison between tertiles and diabetic subjetcs;

(‡) P value for comparison between non-diabetic versus diabetic subjetcs;

(§) p value for interaction analysis between diabetes and T2DM risk alleles.

**Figure 2:**
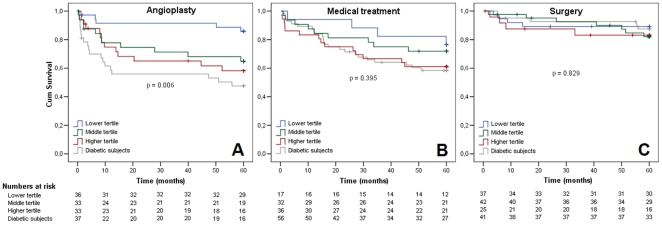
Kaplan-Meier curves for composite cardiovascular end-point in non-diabetic subjects separated by groups according to number of risk alleles tertiles and diabetic individuals, in accordance with the kind of treatment received (A - angioplasty, B - medical therapy or C - CABG). Note for patients submitted to angioplasty, diabetic subjects and higher tertile non-diabetic individuals had higher end-point incidence.

Moreover, resembling the already established behavior of diabetic individuals regarding treatment options for chronic coronary artery disease [23], non-diabetic individuals with more risk alleles showed similar treatment-specific patterns of long-term response. Non-diabetic individuals with higher number of risk alleles, as well as diabetic individuals, submitted to percutaneous coronary interventions presented significantly higher event rates than non-diabetic individuals with low risk, as shown in Figure 2A. The p value for interaction between T2DM risk alleles and treatment received was significant (<0.0001). Information regarding diabetes or genetic risk polymorphims in non-diabetic individuals was not able to long-term stratify individuals submitted to CABG surgery or medical treatment (Figure 2B and 2C).

After multivariate adjustment of a Cox proportional hazards model for several established risk factors for cardiovascular disease, the number of risk alleles were significantly and independently associated with cardiovascular events (p value<0.05). Interestingly, even when fasting glycemia values were included as covariate in the Cox proportional model, it remained significantly associated to composite events incidence (Table 3). In addition, the number of risk alleles was also independently associated to overall mortality (p value = 0.009). We also evaluated 250 patients who completed the 5-years follow up and from whom complete biochemical data was available. Of these, 38 subjects became diabetic at the end of the follow-up period. In the remaining 212 patients (still non-diabetic after 5-years follow-up), the number of risk alleles was also associated with composite cardiovascular end points (p = 0.035) suggesting that the observed association was not entirely due to diabetes emergence during the follow-up period in individuals with more risk alleles.

**Table 3:** Hazard ratio and 95% confidence interval (CI) for composite cardiovascular end-point and mortality according to each risk allele in the entire population and in diabetic and non-diabetic individuals.*

	Non-diabetic subjects			Diabetic subjects			Entire population		
	HR	95%CI	P value	HR	95%CI	P value	HR	95%CI	P value
**Composite Cardiovascular events**									
Number of alleles	1.163	1.02–1.32	**0.022**	1.034	0.87–1.22	0.696	1.113	1.01–1.28	0.033
Number of alleles - no *TCF7L2*	1.125	0.99–1–29	0.084	1.049	0.88–1.26	0.594	1.104	0.99–1.22	0.061

	Non-diabetic subjects			Diabetic subjects			Entire population		
	HR	95%CI	P value	HR	95%CI	P value	HR	95%CI	P value
**Mortality**									
Number of alleles	1.217	1.01–1.46	**0.038**	0.936	0.75–1.16	0.553	1.081	0.94–1.24	0.261
Number of alleles - no *TCF7L2*	1.145	0.95–1.38	0.163	0.925	0.74–1.16	0.493	1.034	0.90–1.19	0.638

(*) adjusted by age, gender, arterial hypertension, BMI, total cholesterol, HDL, LDL, triglycerides, previous MI, smoking and fasting glycemia.

Because it had already been shown that polymorphisms in *TCF7L2* gene were associated with cardiovascular events [16], genotypic information from *TCF7L2* was excluded from analysis to assess whether the positive association of combined information happened only because of *TCF7L2* influence. Even when *TCF7L2* data were excluded, the combined risk alleles remained significantly associated with the composite cardiovascular end-point in non-diabetic individuals (See Figure S1 in Supplemental Material) and with all-cause mortality (p value = 0.036).

**Figure 3:**
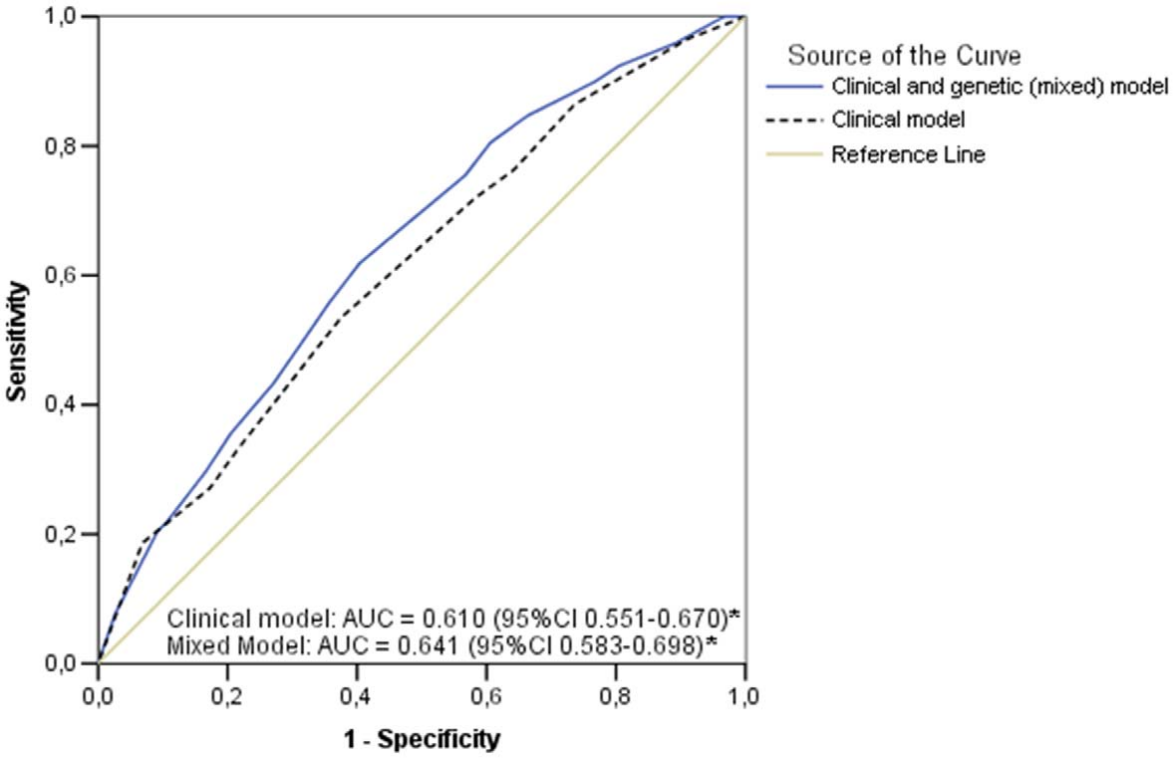
Discriminatory ability for composite cardiovascular events of risk alleles and clinical risk factors in non-diabetic individuals. ROC Curves for cardiovascular events in the MASS-II population. Observe an increased area under ROC curves (AUC or C statistic) after addition of information of combined risk alleles into clinical model for cardiovascular events. (*) Asymptotic significance: P value<0.00001. P values for comparisons between clinical model and mixed model (clinical and genetic model)  = 0.03.

The performance of the predictive risk models can be observed by the ROC curves: if only clinical variables (combination of age, arterial hypertension and previous myocardial infarction) were used to predict cardiovascular events in the MASS-II population, the AUC (C statistics) of this model would be relatively low (AUC = 0.61, p<0.0001). However, the addition of genetic information significantly improved the clinical model's performance (Figure 3): AUC increased to 0.641 (p = 0.03 for comparison between the C statistic of both ROC curves).

## Discussion

The results of our study provide additional information regarding the importance of genetic risk variants for T2DM. Our results extend previous by showing evidence that the utilization of combined genetic information for T2DM risk may still provide valuable information for cardiovascular events prediction. To our knowledge, this is the first study to evaluate the association between combined common genetic variants for type 2 DM in the scenario of major cardiovascular events incidence in chronic coronary artery disease.

Different from other studies where T2DM prediction was the main outcome and the study samples were mainly composed by individuals from the general population, we have explored the potential use of information associated with inherited risk for diabetes, a major cardiovascular risk factor, in the somehow more constrained scenario of multi-vessel chronic coronary artery disease. In this respect, the MASS-II Trial is an exquisite tool by allowing for the test of this hypothesis in a sample of individuals with established chronic coronary artery disease, prospectively followed for 5-years. Our data suggest that risk alleles for T2DM are also able to predict cardiovascular events incidence in non-diabetic individuals. Moreover, the proposed genetic risk model could also stratify non-diabetic individuals in risk strata thus allowing one to identify non-diabetic individuals with similar risk than diabetic individuals. This stratification was above and beyond established cardiovascular risk factors. Interestingly, we were not able to find similar results in diabetic individuals, suggesting that once diabetes is established, cardiovascular risk is high, regardless of the underlying causes of this derangement. Even after adjustment for established risk factors for cardiovascular disease, the risk alleles were independently associated with cardiovascular events (HR for increase of each allele: 1.16, p = 0.022). Similar results were found regarding all-cause mortality after decompounding the end points in non-diabetic individuals. Our data suggest that a subgroup of individuals potentially harboring an increased risk of becoming diabetic could be targeted for more precocious and rigorous preventive efforts, similar to what is advocated for diabetic individuals with coronary artery disease.

Previously, Haffner et al. [24] have demonstrated that incidence rates of myocardial infarction in non-diabetic individuals with prior myocardial infarction were comparable to diabetic subjects without previous myocardial infarction. Interestingly, in our work, individuals with more risk alleles had rates of cardiovascular events comparable to those who had diabetes. In addition, data from the BARI study [25] have found that diabetic patients with multi-vessel coronary artery disease who underwent coronary revascularization therapy with angioplasty had higher event rates than those that underwent CABG or medical treatment. Similarly, in our data, non-diabetic patients in the highest risk strata also had this pattern according to the treatment option they were randomized to. A significant interaction supports the explanation that these two variables do not influence the cardiovascular endpoints in an additive manner, i.e., the simultaneous influence of the type of treatment (PCI, CABG or medical treatment) and risk alleles have consequences on cardiovascular events beyond the simple sum of these variables.

We did not observe any association between number of alleles tertiles and cardiovascular risk factors at baseline in the studied sample. It is thus reasonable to speculate that the observed association between combined T2DM risk alleles and cardiovascular events occurs due to elevations in glycemia, even in a normal, fasting or postprandial, range. Indeed, a recent meta-analysis confirmed the significant association between 2-hour glucose levels after an oral glucose overload and incident cardiovascular events [23]. Coutinho et al. carried out a meta-analysis of 20 studies for a total of more than 90,000 non-diabetics individuals and have found an exponential relationship between the risk of cardiovascular events and both fasting and postload plasma glucose levels. Such relationship was present at even below diagnostic glycemia levels for diabetes, impaired fasting glycemia or impaired glucose tolerance [23]. In addition, systematic evaluation of databases such as deCODE have shown that this relationship between glycemia and cardiovascular risk begins within normal glycemia range, with a linear relationship and shows no indication of a threshold [26]. These data support the hypothesis that even non-diabetic levels of fasting and mainly postprandial hyperglycemia are associated with cardiovascular events incidence and could explain the reason why non-diabetic individuals had similar cardiovascular events than diabetic subjects. In addition, considering that the association of glucose with CVD risk is well established, it would be quite possible that fasting glucose may capture most of the risk conferred by the genetic variants in non-diabetic individuals. Indeed, after including this variable in the model, there was a reduction in the magnitude of effects. Nevertheless, combined T2DM risk alleles remained associated with composite cardiovascular end point and all-cause mortality. In addition, even after exclusion of subjects that became diabetics at end of follow-up period, the association between cardiovascular events and T2DM risk alleles persisted.

Some potential limitations of our study should be acknowledged. Sample size was relatively small. However, even a relatively small sample size of non-diabetic subjects was able to provide a clear understanding of the relative importance of combined genetic information as a predictor of cardiovascular events due to the relatively large effect size of the derived genetic model. On the other hand, there were only a limited number of diabetic individuals and one cannot exclude that this has led to reduced statistical power to detect the association that we were able to describe in non-diabetic individuals. The MASS-II clinical trial was designed for specific purposes and this work was an observational study that used data from MASS-II. Therefore, it is not possible to exclude that residual unmeasured characteristics or confounding factors may influence the results. In addition, considering the specific characteristics of the studied population, the internal and external validation of results may be limited, i.e. to extrapolate the results to population with multivessel CAD with ventricular disfunction or even non-diabetic individuals from the general population further studies will be necessary. Moreover, there was a relatively high proportion of missing data from the initial study population. Nevertheless, there were no differences between groups of individuals included and excluded in the analysis, therefore exclusion of subjects without genotype data probably did not affect the results. To date, there are approximately 30 risk variants associated with T2DM [6], [8] and only 10 polymorphisms were used in the present study. It is possible, therefore, that if other variables were included in risk prediction models, the results would be even more significant. Another limitation is that we did not assess postprandial glicemia at baseline in our study. Thus, it is possible that similar information could be obtained through an oral glucose tolerance test at baseline. As a result, this study is not able to confirm that the findings are a result of hyperglycemia, or postprandial hyperglycemia, even in normal levels. Neither, we can exclude that the genetic effect may be captured by HbA1c or post-prandial/OGTT glycemic values. Nevertheless, it is interesting to note that genetic tests may potentially provide more time for the institution of secondary prevention and do not require to be repeated periodically, characteristics that may allow for the development of more cost-effective management programs for this specific population.

In conclusion, combined information on genetic variants for diabetes risk was associated with major cardiovascular events incidence in non-diabetic individuals with coronary artery disease. In addition, our data suggest that non-diabetic individuals with higher number of T2DM risk alleles had an incidence of cardiovascular events similar to individuals with established diabetes. Other studies are necessary to confirm our results in similar and different populations.

## Supporting Information

Figure S1 **Kaplan-Meier Curves of combined risk alleles without** ***TCF7L2*** **data and composite cardiovascular end points in non-diabetic individuals separated by groups according to number of alles tertiles after 5 years of follow-up.** Even when TCF7L2 data were excluded from analysis, the combined risk alleles remained significantly associated with the composite cardiovascular end-point in non-diabetic individuals. According to number of alleles tertiles, individuals with more risk alleles had higher incidence of cardiovascular events.
(TIF)

Table S1 **Comparison between included and excluded subjects in final analysis.** It is observed that no significant differences between the group of individuals who were included and those who were excluded from analysis. P value for comparison between the groups.
(DOC)

Table S2 **Risk variables, Cox regression analysis and points of Clinical Model and Mixed (Clinical and Genetic Model) for construction of models.** The clinical model that contained age, arterial hypertension and previous myocardial infarction as variables was built by the sum of beta coefficient values from Cox regression multiplied by 10, and rounded to the nearest integer. For example, a 57 years-old individual with hypertension but no previous myocardial infarction and with 13 risk alleles had a score value equal to 10.
(DOC)

Table S3 **Association between genetic factors and type 2 diabetes in the MASS-II Population.** The odds ratios for the risk of type 2 diabetes were calculated with the use of univariate logistic-regression analyses with adjustment for age at participation and sex. The primary genetic models are additive; alternative models are indicated. CI denotes confidence interval, and RAAFF frequency of the risk allele in affected subjects. After Bonferroni's correction for multiple statistical tests (considering a p value more restrictive), no polymorphisms was associated with T2DM.
(DOC)

Table S4 **Combination of Clinical Factors and Risk Alleles in Predicting the Diagnosis of Type 2 Diabetes in the MASS-II Study calculated by multivariate logistic regression.** Only hypertension and combined risk alleles were independently associated with T2DM, after adjustement by multivariate regression. Age, body mass index and risk alleles were included in the model as continuous variables, while hypertension, smoking and sex were included as categorical variables.
(DOC)

Table S5 **Proportion of subjects with T2DM according to genetic risk group (based in tertile of risk alleles).** Odds ratio and p value for comparison between high and low risk groups.
(DOC)
