# Supplementary figures and images for: Genetic Variants of Diabetes Risk and Incident Cardiovascular Events in Chronic Coronary Artery Disease
Source: PLoS One. 2011 Jan 20;6(1):e16341. doi: 10.1371/journal.pone.0016341 (PMC3024434; doi:10.1371/journal.pone.0016341)

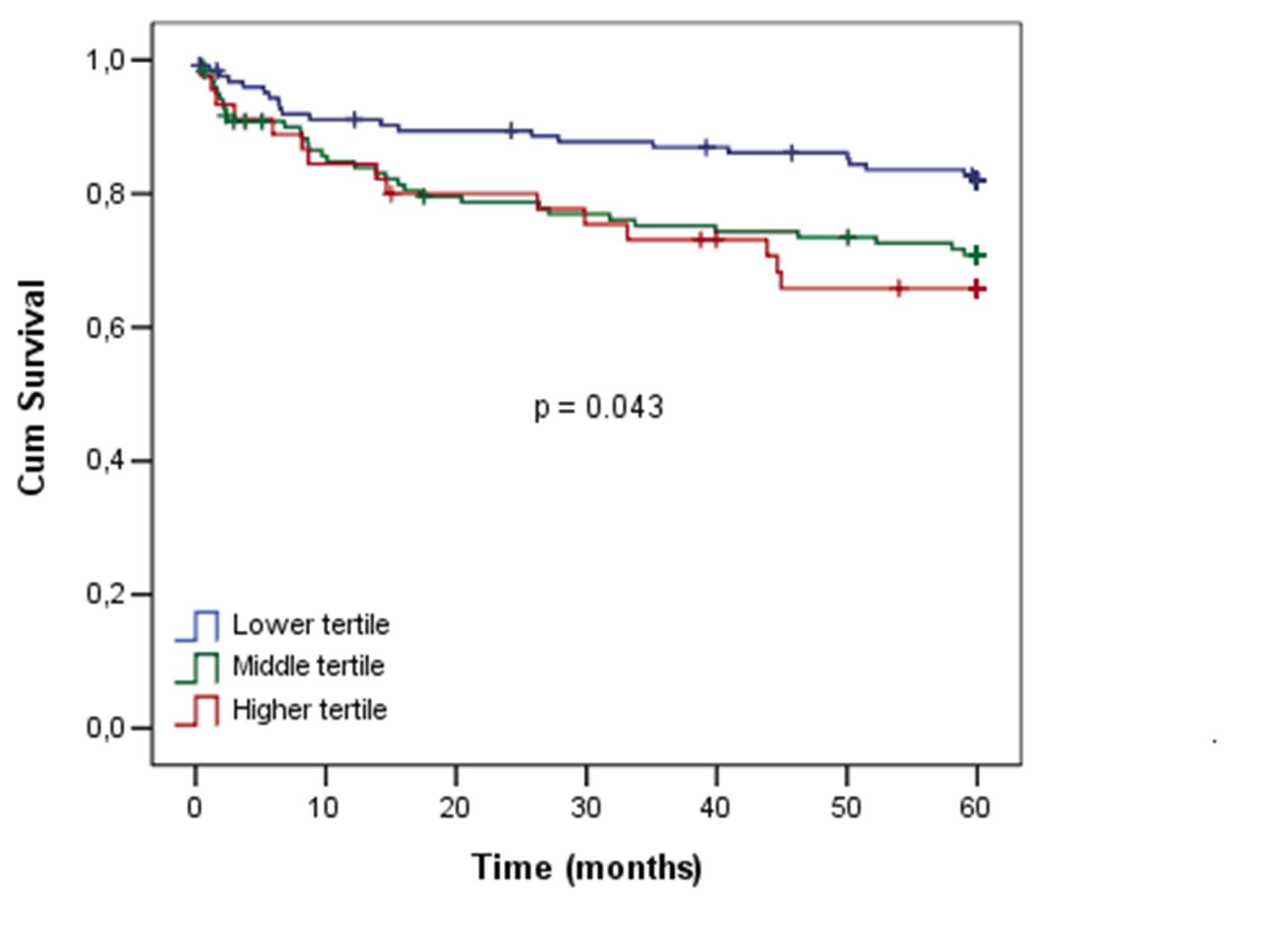

Supplement: Figure S1 — Kaplan-Meier Curves of combined risk alleles without TCF7L2 data and composite cardiovascular end points in non-diabetic individuals separated by groups according to number of alles tertiles after 5 years of follow-up. Even when TCF7L2 data were excluded from analysis, the combined risk alleles remained significantly associated with the composite cardiovascular end-point in non-diabetic individuals. According to number of alleles tertiles, individuals with more risk alleles had higher incidence of cardiovascular events. (TIF) [file pone.0016341.s001.tif]
