# Supplementary material for: Genetic Variants of Diabetes Risk and Incident Cardiovascular Events in Chronic Coronary Artery Disease
Source: PLoS One. 2011 Jan 20;6(1):e16341. doi: 10.1371/journal.pone.0016341 (PMC3024434; doi:10.1371/journal.pone.0016341)
Supplement: Table S1 — Comparison between included and excluded subjects in final analysis. It is observed that no significant differences between the group of individuals who were included and those who were excluded from analysis. P value for comparison between the groups. (DOC) [file pone.0016341.s002.doc]

Table S1 – Comparison between included and excluded subjects in final analysis

|  | **Included**  **(n=425)** | **Excluded**  **(n=186)** | **P value** |
| --- | --- | --- | --- |
| Age (years) | 59.65 ± 9.20 | 60.02 ± 9.11 | 0.647 |
| Gender (male %) | 67.5% | 73.1% | 0.168 |
| BMI (kg/m2) | 27.20 ± 4.15 | 26.82 ± 4.24 | 0.301 |
| Total cholesterol (mg/dl) | 224.07 ± 50.41 | 221.11 ± 40.67 | 0.486 |
| HDL-cholesterol (mg/dl) | 37.21 ± 10.33 | 37.76 ± 10.52 | 0.555 |
| LDL-cholesterol | 148.69 ± 44.83 | 143.58 ± 38.22 | 0.198 |
| Triglycerides (mg/dl) | 194.86 ± 119.31 | 195.70 ± 125.24 | 0.938 |
| Hypertension (%) | 58.8% | 61.0% | 0.083 |
| Smoking (%) | 32.9% | 36.0% | 0.459 |
| Previous MI (%) | 45.4% | 41.6% | 0.751 |
| Diabetes (%) | 31.5% | 30.1% | 0.727 |
