# Supplementary material for: Genetic Variants of Diabetes Risk and Incident Cardiovascular Events in Chronic Coronary Artery Disease
Source: PLoS One. 2011 Jan 20;6(1):e16341. doi: 10.1371/journal.pone.0016341 (PMC3024434; doi:10.1371/journal.pone.0016341)
Supplement: Table S2 — Risk variables, Cox regression analysis and points of Clinical Model and Mixed (Clinical and Genetic Model) for construction of models. The clinical model that contained age, arterial hypertension and previous myocardial infarction as variables was built by the sum of beta coefficient values from Cox regression multiplied by 10, and rounded to the nearest integer. For example, a 57 years-old individual with hypertension but no previous myocardial infarction and with 13 risk alleles had a score value equal to 10. (DOC) [file pone.0016341.s003.doc]

Table S2 - Risk variables, Cox regression analysis and points of Clinical Model and Mixed (Clinical and Genetic Model) for construction of models

| **Variable** | **Groups** | ***β* Coefficient** | **Punctuation**  **(*β* x 10)** |
| --- | --- | --- | --- |
| Hypertension | Hypertension (+) | 0.300 | 3 |
| Age | 55-65yrs  > 65yrs | 0.235  0.696 | 2  7 |
| Previous myocardial infarction | MI (+) | 0.249 | 2 |
| Number of risk alleles | 12-13  > 14 | 0.540  0.743 | 5  7 |
