# Supplementary material for: Genetic Variants of Diabetes Risk and Incident Cardiovascular Events in Chronic Coronary Artery Disease
Source: PLoS One. 2011 Jan 20;6(1):e16341. doi: 10.1371/journal.pone.0016341 (PMC3024434; doi:10.1371/journal.pone.0016341)
Supplement: Table S3 — Association between genetic factors and type 2 diabetes in the MASS-II Population. The odds ratios for the risk of type 2 diabetes were calculated with the use of univariate logistic-regression analyses with adjustment for age at participation and sex. The primary genetic models are additive; alternative models are indicated. CI denotes confidence interval, and RAAFF frequency of the risk allele in affected subjects. After Bonferroni's correction for multiple statistical tests (considering a p value more restrictive), no polymorphisms was associated with T2DM. (DOC) [file pone.0016341.s004.doc]

Table S3 – Association between genetic factors and type 2 diabetes in the MASS-II Population

| Gene | Chromo-some | SNP | Risk Allele | RA_AFF_ | Additive Model | | Alternative Model | |
| --- | --- | --- | --- | --- | --- | --- | --- | --- |
|  |  |  |  |  | Odds Ratio | P value | Odds Ratio | P value |
| *KCNJ11* | 11 | rs5219 | T | 0.33 | 1.003 (0.738-1.364) | 0.984 | Recessive  1.398 (0.744-2.627) | 0.298 |
| *PPARG* | 3 | rs1801282 | C | 0.93 | 1.464 (0.837-2.560) | 0.182 | Recessive  1.444 (0.819-2.548) | 0.204 |
| *TCF7L2* | 10 | rs7903146 | T | 0.47 | 1.511 (1.069-2.135) | 0.019 | Recessive  2.039 (1.106-3.760) | 0.022 |
| *SLC30A8* | 8 | rs13266634 | C | 0.76 | 1.137 (0.818-1.582) | 0.444 | Recessive  1.238 (0.816-1.880) | 0.315 |
| *HHEX* | 10 | rs1111875 | G | 0.68 | 1.274 (0.946-1.716) | 0.111 | Recessive  1.420 (0.939-2.147) | 0.096 |
| *CDKAL1* | 6 | rs7754840 | C | 0.37 | 1.198 (0.885-1.622) | 0.243 | Dominant  1.291 (0.850-1.963) | 0.231 |
| *IGF2BP2* | 3 | rs4402960 | T | 0.39 | 1.237 (0.899-1.702) | 0.191 | Recessive  1.419 (0.744-2.707) | 0.289 |
| *CDKN2A/B* | 9 | rs10811661 | T | 0.85 | 1.307 (0.880-1.941) | 0.184 | Recessive  1.406 (0.894-2.212) | 0.141 |
| *CDKN2A/B* | 11 | rs9300039 | C | 0.90 | 1.095 (0.684-1.753) | 0.705 | Recessive  1.214 (0.718-2.051) | 0.470 |
| *FTO* | 16 | rs8050136 | A | 0.40 | 1.010 (0.743-1.374) | 0.948 | Dominant  1.167 (0.756-1.801) | 0.486 |

The odds ratios for the risk of type 2 diabetes were calculated with the use of univariate logistic-regression analyses with adjustment for age at participation and sex. The primary genetic models are additive; alternative models are indicated. CI denotes confidence interval, and RAAFF frequency of the risk allele in affected subjects. After Bonferroni´s correction for multiple statistical tests (considering a p value more restrictive), no polymorphisms was associated with T2DM
