# Supplementary material for: Genetic Variants of Diabetes Risk and Incident Cardiovascular Events in Chronic Coronary Artery Disease
Source: PLoS One. 2011 Jan 20;6(1):e16341. doi: 10.1371/journal.pone.0016341 (PMC3024434; doi:10.1371/journal.pone.0016341)
Supplement: Table S4 — Combination of Clinical Factors and Risk Alleles in Predicting the Diagnosis of Type 2 Diabetes in the MASS-II Study calculated by multivariate logistic regression. Only hypertension and combined risk alleles were independently associated with T2DM, after adjustement by multivariate regression. Age, body mass index and risk alleles were included in the model as continuous variables, while hypertension, smoking and sex were included as categorical variables. (DOC) [file pone.0016341.s005.doc]

Table S4 – Combination of Clinical Factors and Risk Alleles in Predicting the Diagnosis of Type 2 Diabetes in the MASS-II Study calculated by multivariate logistic regression

|  | **Odds Ratio (95%CI)** | **P Value** |
| --- | --- | --- |
| **Additive Model** |  |  |
| Male sex  Age, per 1 year  Smoking  Hypertension  Increased body-mass index, per 1 kg/m^2^  Combination of risk alleles | 1.068 (0.677- 1.684)  1.013 (0.988- 1.038)  1.169 (0.722- 1.892)  **1.674 (1.069- 2.622)**  1.032 (0.980- 1.087)  **1.178 (1.056- 1.314)** | 0.778  0.311  0.525  **0.024**  0.232  **0.003** |
