# Supplementary material for: Genetic Variants of Diabetes Risk and Incident Cardiovascular Events in Chronic Coronary Artery Disease
Source: PLoS One. 2011 Jan 20;6(1):e16341. doi: 10.1371/journal.pone.0016341 (PMC3024434; doi:10.1371/journal.pone.0016341)
Supplement: Table S5 — Proportion of subjects with T2DM according to genetic risk group (based in tertile of risk alleles). Odds ratio and p value for comparison between high and low risk groups. (DOC) [file pone.0016341.s006.doc]

Table S5 - Proportion of subjects with T2DM according to genetic risk group (based in tertile of risk alleles)

|  | **Diabetes (%)** | **Odds Ratio (95%CI)** | **P value** |
| --- | --- | --- | --- |
| **Genetic groups (according to nº of alleles)** | | |  |
| Low risk (<11) | 26.3% |  |  |
| High risk (>14) | 44.2% | 2.214 (1.273-3.852) | 0.0049 |
